# CAH Newborn Screening in India: Challenges and Opportunities

**DOI:** 10.3390/ijns6030070

**Published:** 2020-08-27

**Authors:** Aashima Dabas, Meenakshi Bothra, Seema Kapoor

**Affiliations:** Department of Pediatrics, Maulana Azad Medical College and Lok Nayak Hospital, New Delhi 110002, India; dr.aashimagupta@gmail.com (A.D.); meenakshibothra@gmail.com (M.B.)

**Keywords:** newborn screening, congenital adrenal hyperplasia, CAH

## Abstract

Congenital adrenal hyperplasia (CAH) is a common treatable disorder which is associated with life-threatening adrenal crisis, sexual ambiguity, and/or abnormal growth if undiagnosed. Newborn screening is a cost-effective tool to detect affected babies early after birth to optimize their treatment and follow-up. Newborn screening however is in its nascent stage in India where it is not yet introduced universally for all babies. The following review briefly highlights the challenges (e.g., lack of universal screening, healthcare resources) and opportunities (e.g., reduction in morbidity and early correct gender assignment in females) associated with newborn screening for CAH in a large Indian birth cohort.

## 1. Introduction

Congenital adrenal hyperplasia (CAH) refers to a group of autosomal recessive disorders caused by inherited defects in steroid biosynthesis. It is most commonly caused by a deficiency of the enzyme 21 alpha hydroxylase, leading to the deficiency of mineralocorticoids and glucocorticoids and excess of sex steroids. The disease presentation may be classical, as salt-wasting (SW) or simple virilizing (SV), and non-classical. Classical CAH is most readily discernible when it presents with virilization in girls, but it may be missed in boys, who may manifest with life-threatening adrenal crisis within the first few weeks of life [1]. 

### Newborn Screening (NBS) for CAH

In 2002, the Joint Lawson Wilkins Pediatric Endocrine Society/European Society for Pediatric Endocrinology Working Group recommended biochemical screening for classical CAH in the newborn period to reduce associated morbidity and mortality [2]. Newborn screening (NBS) for CAH is performed by measuring 17-hydroxyprogesterone (17OHP). This is traditionally measured by radioimmunoassay, enzyme-linked immunosorbent assay or time-resolved fluoroimmunoassay. In an effort to reduce the high false positive rate associated with immunoassay screening for CAH, the 2018 Endocrine Society Clinical Practice Guidelines recommend a second-tier screen by liquid chromatography tandem mass spectrometry (LC/MS-MS) [3].

The levels of 17OHP are influenced by maturity, stress, maternal steroid administration and age. Cord blood is easier and non-invasive, but is not recommended for CAH screening as the steroid surge at birth results in significantly higher 17OHP levels which can be difficult to interpret [4,5]. Capillary blood samples (usually collected later by heel prick onto collection paper) are ideal for measurement of 17OHP for NBS. They also provide an opportunity to screen for other disorders of metabolism in the same sample [5,6]. In a study on newborn screening of 3080 babies from Southern India, prematurity significantly increased the mean 17OHP values from 4.86 ± 2.47 to 8.97 ± 7.43 ng/mL and the median 17OHP value from 4.5 to 6.3 ng/mL (measured on competitive immunoassay) [7]. Investigators from different regions of the World have found that sex, mode of delivery as well as seasonality affect 17OHP values [8,9]. Another study from India comparing the 17OHP levels (radioimmunoassay) in sick vs. healthy neonates demonstrated that gestational age, birth weight, and Apgar score were negatively correlated, whereas stress factor, mode of delivery and use of antenatal steroids in mothers were positively correlated with 17OHP levels [10].

India’s high annual birth rate of 17.8 births per 1000 results in the birth of over 4 million newborns per year, of which over 277 thousand are home births. Screening such a large cohort would not only need a robust newborn screening program, but also some mechanism to ensure that the babies born at their homes are not missed [11]. In medical facilities with high turnover, babies are usually discharged within 24–48 h of delivery, thereby increasing the chances of inadvertently missing these babies if screened 48 h after birth. The sample collection time for newborn screening for CAH is, therefore, a balance between practical considerations about coverage, the best time to assess 17OHP level and the need to have results early enough to minimize the risk of salt-wasting adrenal crisis. Therefore, we suggest the sample for NBS to be taken at least 24 h later, but may be taken up to day 7 of life, using heel-prick [5]. Preterm babies should be screened at 2 weeks and 4 weeks of age or at discharge from NICU, to decrease the risk of missing a case of CAH [3]. In addition, there should be close monitoring of electrolyte imbalance, blood glucose and blood pressure of babies in NICU.

There may be high false positive rates with the first-tier immunoassay due to cross-reactivity and physiological changes in the concentration of 17OHP during the first few days of life, especially in preterm neonates [12]. Screening specificity can be improved with second-tier testing; for example, LC/MS-MS of steroids including 17-hydroxyprogesterone, 21-deoxycortisol, androstenedione, and cortisol [13]. At present, a LC/MS-MS facility is not available for most screening programs in India. However, it is planned to assign a centralized laboratory for confirming screen positive samples in different districts. Infants positive for the newborn screen test should be referred to a pediatric endocrinologist for evaluation. A confirmatory diagnosis may be performed by cosyntropin stimulation test. Molecular testing is an available option for confirmatory diagnosis in screen positive individuals. It will also help in offering prenatal diagnosis in the subsequent pregnancies. However, cost constraints and limited availability remain major hurdles to its current use in the Indian context.

## 2. Disease Burden in India and Need of NBS

The overall disease burden of CAH in India is not known but is likely to be significant. CAH remains the most common cause of female ambiguity and primary adrenal failure in Indian series underscoring the magnitude of the problem [14,15,16]. Most affected newborns succumb to adrenal crisis in their first few weeks of life or early infancy in the absence of a confirmed clinical diagnosis [17,18]. In an Indian report by Rajendran et al., 8 out of 22 (36.4%) unscreened babies who presented with features of salt-wasting CAH in the neonatal period died during infancy, at a median age of 3 months (0.2–13 months) [19]. In a study by Miati et al., there were just 10 males out of a total of 45 children with classical CAH seen at a tertiary clinic, of which, 2 males were noted to have sustained cognitive impairment as a result of multiple episodes of severe hyponatraemic encephalopathy prior to diagnosis. The skewed sex ratio suggests that the affected males with SW-CAH may have died undetected, and that the burden of disease in India is even greater than reported [20]. Virilized female infants with SV-CAH and affected children of both sexes with precocious puberty may not be brought to medical attention for the fear of social stigma, resulting in delayed diagnosis [21].

The incidence and prevalence of CAH in India is not known. However, CAH qualifies for screening in India, as per the Wilson and Jungner criteria as it is seen frequently in our country, is treatable when diagnosed early and might result in irreversible sequelae if left untreated [22]. However, newborn screening is still not being routinely done in India. It has been done intermittently, in project mode, in various studies. Table 1 shows the details of newborns detected to have CAH on newborn screening by various investigators from India. In a multicentric study of the 104,066 newborns screened for CAH, 18 infants (16 SW, 2 SV) were confirmed to have CAH, suggesting a collective incidence of 1 in 5762. The study showed marked regional differences, with the prevalence being 1 in 2036 in Chennai, 1 in 7608 in Delhi and 1 in 9983 in Mumbai [23]. Other recent studies from India, found the incidence of CAH to be 1 in 2800 in South India [24] and 1:6334 in North India [4]. The differences in incidence of CAH reported from India may be because of the variations in technique used, different cut-offs, different disorder definition and/or different populations screened. The incidence figures reported from India are higher than those reported from European countries [25]. This could be due to the high consanguineous marriage rate (1–30%), especially in certain population groups, resulting in higher incidences of recessively inherited diseases. Population stratification into different castes and endogamous mating could also be a contributor [26].

With the Prenatal Diagnostic Act in place in India, identification of gender in the prenatal period is not permitted. The birth of a baby with sexual ambiguity comes with a considerable social stigma for the family. Unless the family is able to conceal genital ambiguity, affected babies are at risk of being whisked away and illegally adopted by Hijra (transgender groups). In India, gender reassignment from ‘male’ to a ‘female’ may be difficult for the affected families for fear of stigmatization [27]. The process of registering a change of gender of the baby in the birth certificate can be complex and tiring for the families within the legal framework. Moreover, where female feticide poses an additional danger to the survival of a girl, the change of gender from ‘male’ to ‘female’ may not be welcomed in many Indian families, even in the 21st century. The clinicians should therefore assist the parents to assign the biological gender as the gender of rearing in those presenting with ambiguity, as also recommended by most consensus groups [28]. Newborn screening informs early and accurate assignment of biological gender and is of high societal importance in India.

Among an Indian cohort of81 children (32 boys, 49 girls) with congenital adrenal hyperplasia due to 21 hydroxylase deficiency, two-thirds (57) had salt-wasting and the remaining had simple virilizing type. Twenty-five (31%) of these children had short stature and 45 (55.6%) had growth velocity below the reference range [29]. CAH is not a target disorder for NBS in Great Britain. A two-year nationwide CAH surveillance study in Great Britain reported 144 cases of childhood CAH, of which 86 (59.7%) presented in infancy (37 simple virilization, 30 salt-wasting, 12 affected siblings, 8 others). Fifty-eight subjects presented later at median age of 5.9 years with precocious puberty in the majority (66%) and genital virilization and affected sibling in 14% each [30]. It is still possible that a few additional children with mild disease were missed in this cohort. Similar findings are echoed in a retrospective case series from India [31], where precocious puberty and growth failure were reported in most children. CAH is the most common cause of female ambiguity which presents during adolescence [14,16]. The pubertal changes and advanced skeletal age are irreversible, thus underscoring the need of NBS for timely detection and management of affected children.

In a cohort study at British Columbia Children’s Hospital, the median age at diagnosis was 5 days (range, 0–30 days) and 6 days (range, 0–13 days) in unscreened and screened neonates, respectively. However, the cost of care was USD 33,770 per case in unscreened vs. USD 17,726 in screened newborns emphasizing the cost-effectiveness of CAH-NBS program [32]. A cost-benefit analysis is currently not available from India.

## 3. Challenges

### 3.1. NBS—Current Situation in India

In India, 10% of deliveries are attended by untrained health personnel and only one-third of babies receive a health check-up by trained health-personnel within two days after birth [35]. There is lack of human resource with expertise in childcare and infrastructure for managing sick babies, which, coupled with above observations greatly increase the odds of missing SW and SV-CAH affected babies. Health is a state subject in India which implies that the budget, human resource, logistics and microplanning of any NBS program has to be decentralized and mapped for each state. The current allocation of budget for health in India is also dismal at a meager 1.15% of GDP [36].

As 17OHP levels vary significantly with gestational age, the cut-offs for NBS should be gestational age-specific. However, this may be a concern in cases where pregnancy may be unrecorded and accurate estimation of gestational age is unavailable. The very high proportion of pregnant Indian women with little antenatal care, and that birth weight of Indian babies tend to be lesser than the Western babies for comparable gestational age, underscore the need of local birth weight-based cutoffs. In such situation, birth weight-based cut-offs for 17OHP may be used based on recently compiled data from Indian cohort [5]. Similar weight-based cutoffs were being used in few European countries and Japan [25].

Gender assignment in India is performed immediately after the birth of a baby. This is a major concern for families with the birth of a virilized baby where male gender becomes the default gender within the patriarchal Indian culture. A study from southern India reported incorrect gender assignment in 6/24 (25%) girls, which was correctly reassigned in 5 babies within 1 month of birth, but at six years in the sixth patient [37]. A large dataset of 62 affected CAH patients (6 males) showed ambiguity in 56 (90%), with wrong gender assignment in five affected females [20].

Limited access to specialty endocrinology services and non-availability of appropriate treatment and follow-up facilities remain additional challenges for the babies with CAH, especially from the rural and remote areas. Hydrocortisone, being the drug of choice, has limited availability in remote parts of India, where patients may resort to use of alternative agents like prednisolone or dexamethasone. Chandigarh began newborn screening for CAH for all institutional deliveries in 2007, one of the first states in India to do so [4]. However, the recall and false positive rates were reported to be high in this state, similar to other Asian countries [4,38]. The ‘recall rate’ means the proportion of babies who the program is able to recall for review following a positive screen, as shown in Table 1. The factors that contribute to poor response to recall include illiteracy, limited healthcare delivery in outreach areas and unawareness of the need for repeating NBS tests. Comprehensive newborn screening is being offered to all births in Kerala, a state in southern India. The NBS program launched in Kerala in 2012, screens for four disorders including congenital hypothyroidism, CAH, Glucose 6 phosphate dehydrogenase deficiency and Galactosemia. Since then, the program has grown to screen more than 140,000 births per year in over 90 government hospitals of Kerala [39].

The lack of NBS across India, even for congenital hypothyroidism, which is a disorder that most developing programs would start with, underscores the need for increasing awareness about the utility of newborn screening in the Indian population. There is also limited public awareness in India about the need to test apparently healthy newborns for life-endangering congenital disorders like CAH. A funded publicity program with contributions from all healthcare professionals (pediatricians, midwives, obstetricians, geneticists and others) will be needed to create this awareness and subsequent agreement to testing and follow up. NBS will have to be eventually integrated with existent maternal and child health programs to ensure universal coverage. Medical colleges and tertiary level hospitals can take the lead in offering NBS services which will later expand their services to cover outreach areas [36].

### 3.2. Communicating with Families

A diagnosis of CAH is a lifelong identification which should be communicated to the family in a responsive manner. The communication should be done in a more sensitive manner with parents of affected female babies who presented with virilization. The correct gender of the baby should be communicated to the parents ensuring that privacy is maintained. The family is also counseled to maintain confidentiality of the diagnosis to decrease the likely risk of the baby being taken away by eunuch (*hijra*) groups, which are communities of transgender individuals, who are usually considered as social outcastes. This community not only lacks gender recognition and sexual expression, but also has to face limited employment, poor living conditions as well as discrimination on various fronts.

Screening of other members in the family should be performed through a detailed history including three generation pedigree especially in countries which have a high number of consanguineous marriages. In a study from Syria, 14.6% of affected cases had positive family history with a family history of similar complaint in another 18% [40]. An estimated 1–30% marriages are consanguineous in India across different communities [20,41]. Added to this, is the burden of endogamity which increases the probability of manifestation of genetic disorders.

### 3.3. Healthcare Services (Including Patient Education and Support Groups) in India

There exists an inequitable distribution of healthcare services in India with marked geographical variations which limit access to tertiary level health care services in some areas. The doctor to patient ratio is poor due to the lack of trained and qualified doctors. Thus, the parents of affected children should be educated about adrenal insufficiency and possible risk of adrenal crisis. They should be educated to identify an acute illness at home and increase the dose of replacement steroids to prevent adrenal crisis. An identity card should be carried by all CAH-affected children which should mention the identification details, diagnosis of the child and emergency contact numbers. The card can also mention the need for stress doses of steroids in acute illness which can help in acute management of the child in the remotest corner of the country. The Indian Society for Pediatric and Adolescent Endocrinology (ISPAE) has developed a patient education booklet in English and Hindi (local language) [42]. A similar booklet has been developed by Department of Science and Technology, India in local language (Hindi) for improving patient information. These booklets are shared with the parents at diagnosis which helps them to understand the nature of disease and cope with the stress.

## 4. Opportunities

The National Health Policy of India, 2017, promotes the provision of quality health care with dynamic cooperation between national and international partners and emphasizes promoting preventive health care, early detection and management of childhood problems [43]. Universal newborn screening for treatable genetic disorders including congenital hypothyroidism and congenital adrenal hyperplasia, however, has still not seen the light of the day in India. Newborn screening in India is only done in a project mode, in specific medical facilities, cities or states, intermittently. The known roadblocks include lack of awareness among healthcare providers, non-institutional deliveries in a significant proportion (22%) of women, logistic and financial issues to cater to a huge birth cohort of 4 million babies annually, lack of sensitization among policy makers and governance issues. To promote the early detection and management of childhood disorders, the Government of India launched the Rashtriya Bal Suraksha Karyakram (RBSK) in 2013, which aimed to screen over 270 million children from 0 to 18 years for four Ds—Defects at birth, Diseases, Deficiencies, and Development Delays including Disabilities. The Delhi government recently launched its flagship health program under the vision of RBSK called ‘Mission NEEV’ (Neonatal Early Evaluation Vision), to provide universal newborn screening for congenital hypothyroidism and congenital adrenal hyperplasia, early diagnosis of visible birth defects, critical congenital heart diseases and hearing loss. The program is being launched at 31 major birthing facilities of the city and will later be expanded to all delivery points and other birthing facilities [44]. In addition to universal NBS for CAH, it is also important to ensure the appropriate management and long term follow-up of individuals diagnosed with CAH. The potential for working with international partners and funding agencies to establish screening, increase awareness about the need of screening for CAH, patient group advocacy with politicians, starting screening even in small hospitals with patients who can afford additional testing, establishing a dried blood spot diagnostic service for patients at risk capacity building, and dried blood spot technology development in hospitals where this does not exist but which might offer screening in the future, all offer exciting future possibilities. The dried blood spot service can also be used for the monitoring of serial samples in individuals with CAH.

## 5. Conclusions

NBS offers us hope for the timely detection of CAH. It will help in the timely instituting of therapy to prevent medical and psychosocial complications to improve disease outcomes and decrease the associated morbidity. The logistics for NBS-CAH are limited at present in the Indian context, primarily because of a lack of resources for newborn screening generally, but also because of the limited opportunities to reduce false positive tests by steroid profiling. However, considering not only the mortality and morbidity of severe CAH but also the sizeable population of this treatable condition in India, CAH may be an essential disorder to be screened and included in the core panel of NBS as this is introduced.

## Figures and Tables

**Table 1:** Published literature on newborn screening for CAH in India.

Author	Year of Publication	Number of Babies Screened	Results	Method Used	False Positivity	Incidence of CAH
Kommalur et al. [33]	2019	41,027	13 babies screen positive, out of which 11 babies could be recalled	17-OHP level by time resolved fluoroimmunoassay	1/11 (9%)	1 in 4102
Verma et al. [34]	2019	13,376	15 babies screen positive; All 15 recalled, 5 found to be true positive	17-OHP level by time resolved fluoroimmunoassay	10/15 (66.7%) were false positive	1 in 2500
ICMR Task force [22]	2018	104,066	142 positive on initial screening; 80% babies could be recalled; 18 infants were confirmed to have CAH (16 salt wasting and 2 simple virilizing)	17-OHP level by time resolved fluoroimmunoassay	96/114 (84.2%) babies false positive	1 in 5762
Anandi et al. [7]	2017	3080 (retrospective analysis)	Retesting done in 82 babies as 17-OHP levels were above cut-off	17-OHP level by competitive enzyme immuno assay	82/82 (100%) babies false positive	-
Kumar et al. [23]	2015	11,200	15 positive on initial screening.	17-OHP level by time resolved fluoroimmunoassay	11/15 (73.3%) babies were false positive	1 in 2800
Kaur et al. [4]	2010	6813	22 positive on initial screening; 7/22 babies could not be recalled;	17-OHP level by time resolved fluoroimmunoassay	13/14 (92.8%) babies false positive	1 in 6813
